# Genetic and proteomic approaches to identify cancer drug targets

**DOI:** 10.1038/bjc.2011.543

**Published:** 2011-12-13

**Authors:** G Roti, K Stegmaier

**Affiliations:** 1 Department of Pediatric Oncology, Dana-Farber Cancer Institute and the Division of Pediatric Hematology/Oncology, Children's Hospital Boston, Harvard Medical School, Boston, MA 02215, USA; 2 The Broad Institute of Harvard University and Massachusetts Institute of Technology, Cambridge, MA 02142, USA

**Keywords:** drug discovery, protein target identification, chemical genomics, chemical proteomics, small-molecule library screening, cancer drug targets

## Abstract

While target-based small-molecule discovery has taken centre-stage in the pharmaceutical industry, there are many cancer-promoting proteins not easily addressed with a traditional target-based screening approach. In order to address this problem, as well as to identify modulators of biological states in the absence of knowing the protein target of the state switch, alternative phenotypic screening approaches, such as gene expression-based and high-content imaging, have been developed. With this renewed interest in phenotypic screening, however, comes the challenge of identifying the binding protein target(s) of small-molecule hits. Emerging technologies have the potential to improve the process of target identification. In this review, we discuss the application of genomic (gene expression-based), genetic (short hairpin RNA and open reading frame screening), and proteomic approaches to protein target identification.

The last decade has seen dramatic change in approaches to biomedical discovery through the advent of massively parallel sequencing technologies to sequence genomes, the ability to characterise the transcriptome and an improved ability to evaluate the proteome. Nowhere, perhaps, has this change been more readily apparent than in cancer-based discovery where cancer-promoting targets are being identified at an ever-increasing pace. An important challenge now is how to best exploit these new capabilities for therapeutic benefit. In this review, we focus on one area of increasing need: the development of new approaches to identify the direct protein-binding targets of small molecules. Specifically, we focus on the application of chemical genomics, genetic-based screening, and chemical proteomics to identify the critical protein targets of drugs and discuss illustrative examples of each (Figure 1).

## Advances in phenotype-based screening: new chemical discoveries but new challenges

With the demonstration of clinical efficacy for targeted agents, such as all-trans-retinoic acid in *PML-RARα*-rearranged acute promyelocytic leukaemia, trastuzumab treatment for *HER2*-amplified breast cancer, and imatinib therapy for *BCR–ABL*-rearranged chronic myelogenous leukaemia, there has been a sea change in cancer-based drug discovery (Capdeville *et al*, 2002; Sanz, 2006; Hudis, 2007; Lo-Coco *et al*, 2008). Currently, the majority of cancer drug discovery efforts in the pharmaceutical industry are target based. Although there is a strong rationale for target-based therapies, such as improved tumour cell specificity and decreased normal cell toxicity, there are limitations to target-based screening approaches. First, target-based screening typically involves *ex cellulo* assays, which may not recapitulate cellular complexity. Second, many validated cancer targets, such as transcription factor abnormalities, have been difficult to ‘drug’ using standard target-based screening. High-throughput assays to measure DNA–protein or protein–protein interactions have proven difficult to develop. Third, there are many desired state changes (e.g., differentiation) for which a validated protein target has not yet been identified. In response to these challenges, new approaches to phenotypic screening have been developed. One area of progress has been the application of high-content imaging to enable fine-grained phenotypic measurements from single cells and kinetic studies of living cells in response to small-molecule perturbation (Giuliano *et al*, 1997, 2003; Carpenter, 2007).

Another new approach to phenotypic screening leverages the assessment of gene expression signatures as surrogates for different biological states. One implementation of this approach is gene expression-based high-throughput screening (GE-HTS) (Stegmaier *et al*, 2004). Genome-wide expression-based microarrays are used to define a set of genes distinguishing two different biological states, and the signature is refined to include up to 500 genes, which can be measured in 384-well format. One successful detection system has used ligation-mediated amplification and a fluorescent bead-based technology (Peck *et al*, 2006), although one can envision the implementation of other technologies such as Nanostring or RNAseq. Using this method, a small-molecule library is screened for chemicals that induce a change from the ‘state A’ expression signature to that of the ‘state B’. Gene expression-based high-throughput screening has been applied to the identification of compounds inducing tumour cell differentiation, modulators of transcription factor abnormalities, inducers of fetal haemoglobin, inhibitors of androgen receptor activation, and modulators of mitochondrial oxidative-phosphorylation physiology (Stegmaier *et al*, 2005; Hieronymus *et al*, 2006; Wagner *et al*, 2008; Corsello *et al*, 2009; Hahn *et al*, 2009; Bradner *et al*, 2010).

Although alternative screening approaches may address some of the current challenges in small-molecule discovery, such as targeting intractable cancer-promoting proteins, or identifying modulators of complex state switches in the absence of target knowledge, as in the case of more traditional phenotypic-based screening, they engender the problem of identifying the protein target of the small-molecule hit. Target identification is critical to follow-up medicinal chemistry efforts, to identify predictors of response to therapy, and to understand mechanisms of resistance. Affinity chromatography is one standard approach used to identify the protein targets of small molecules. This approach is limited, however, by the requirement of small molecules with both high affinities for their targets and for high expression of the protein target of interest. Moreover, the generation of affinity matrices, which immobilise a derivative of the small molecule but also retain the original activity of the molecule, often require significant optimisation. New genomic, genetic, and proteomic approaches, discussed below, should improve the pipeline for protein target identification of chemical hits emerging from cell-based, phenotypic screens.

## Gene expression-based solutions to protein target identification

Transcriptional profiling of chemically or genetically manipulated cellular systems can be used to connect a compound of interest with its protein target. One of the first proof-of-concept studies was performed in *Saccharomyces cerevisiae* (Hughes *et al*, 2000). Hughes *et al* generated a compendium of expression profiles of genetic mutations or drug treatments affecting *Saccharomyces cerevisiae* cells. They demonstrated that different mutants or chemical treatments affecting similar cellular processes induce similar expression profiles, suggesting that the compendium could be used to characterise the mechanism of pharmacological perturbations by using gene expression pattern-recognition algorithms.

Expanding on this idea, Lamb *et al* developed the Connectivity Map (C-Map), the first mammalian compendium of chemically perturbed transcriptional profiles, as an *in silico* tool for signature-based small-molecule and target discovery (Lamb *et al*, 2006; Lamb, 2007; Palchaudhuri and Hergenrother, 2011). The C-Map is a reference collection of genome-wide transcriptional expression data from cultured human cancer cells (breast cancer MCF7, prostate cancer PC3, leukaemia HL60, and melanoma SKMEL5 cell lines) profiled on the Affymetrix (Santa Clara, CA, USA) high-throughput array platform. In its first public release (Build 01), the C-Map profiled 164 bioactive small molecules at 6 h, with most compounds profiled at 10 *μ*M. Each treatment instance was defined relative to control cells treated with vehicle and grown in the same plate. For each treatment in the C-Map, the 22 000 genes were rank ordered based on their differential expression relative to the batch control. The C-Map user defines a query signature associated with a particular condition of interest, which is then assessed for connection with each of the profiled compounds in the reference collection using a rank-based pattern matching algorithm. The C-Map is available on a publicly accessible website www.broadinstitute.org/cmap, which also includes meta-data (chemical name, concentration, cell line, and batch) and analytical tools, provides links for each compound to a vendor and to Chembank (a database for structures and synonyms), and displays the Anatomical Therapeutic Chemical classification assigned by the World Health Organisation to drug substances. A new users' tutorial is available at the same web site in the ‘Help’ section. This guide is intended to take users through the basic steps in executing and interpreting a C-Map query. It also includes definitions of key C-Map-related terminology. The second build of the C-Map (Build 02) is publicly available and includes the profiling of 1309 discrete small molecules with raw data cel files available for download enabling user data-mining of the full compendium of expression profiles. The next iterations of C-Map will also include genetic perturbation, such as short hairpin RNA (shRNA) and open reading frames (ORFs).

To illustrate the potential of C-Map to connect a small molecule with its protein target, we first discuss a study, which integrates a GE-HTS screen for lead molecule discovery with the C-Map for protein target identification. Hieronymus *et al* (2006) sought to identify chemical modulators of androgen receptor signalling in prostate cancer. Given the paucity of available approaches to identify modulators of androgen receptor signalling, a GE-HTS approach was used. First, a gene expression signature was defined by identifying the genes whose expression distinguishes the androgen activation versus deprivation states using Affymetrix expression microarray profiling and adapted to the GE-HTS assay. A total of 2500 compounds were then screened for the ability to modulate a 27-gene signature in the presence of synthetic androgen in the prostate cancer cell line LNCaP. The natural products celastrol and gedunin were identified and confirmed to strongly induce the androgen deprivation signature. As a result of the lack of knowledge of the protein targets of gedunin and celastrol, the investigators leveraged the C-Map to identify their mechanism of action. Genome-wide expression profiles of gedunin- and celastrol-treated LNCaP cells were generated and used to query the C-Map. The gedunin and celastrol profiles were strongly connected to the gene expression profiles of multiple, structurally distinct HSP90 inhibitors leading to the hypothesis that these agents were themselves HSP90 inhibitors. Indeed, both molecules were demonstrated to inhibit HSP90 activity and HSP90 clients by a mechanism that is distinct from that of existing HSP90 ATP-binding pocket inhibitors.

Multiple examples now exist of the application of the C-Map to connect small-molecule modulators with their mechanisms of action and/or protein targets. Two recent examples connect small-molecule hits emerging from independent cancer cell-based screens with intracellular iron depletion (Coombs *et al*, 2011; Gullbo *et al*, 2011). Gullbo *et al* (2011) screened 3000 compounds in primary ovarian carcinoma cultures for those inducing cytotoxicity and identified the compound CD02750 (VLX50) and structurally related molecules as top hits. After confirming broad spectrum activity in a panel of patient-derived cancer cells, a drug-specific signature was identified using genome-wide expression profiling and used to query the C-Map. The VLX50 signature showed the strongest correlation with several iron chelators and also showed a significant enrichment with genes induced by hypoxia and hypoxia-inducible factor 1 *α* (HIF-1*α*) by gene set enrichment analysis (GSEA), also consistent with iron chelation (Subramanian *et al*, 2005). The investigators experimentally confirmed that VLX50 decreases iron concentration in MCF-7 cell cultures and that VLX50 drives the expression of genes associated with HIF-1 signalling.

In an independent screen for compounds that inhibits Wnt/*β*-catenin pathway using a cell-based luciferase reporter, Coombs *et al* (2011) also connected the small-molecule candidates emerging from their screen to iron chelation via the C-Map. From 50 000 synthetic compounds, a family of 8-hydroxyquinolone derivatives was identified. The lead compound *N*-((8-hydroxy-7-quinolinyl)(4-methylphenyl)methyl)benzamide (HQBA) was demonstrated to inhibit growth in both Wnt-initiated and Wnt/*β*-catenin-dependent MMTV-Wnt1-transgenic mice and in MMTV-PyMT mice with no evidence of activation of the Wnt-catenin pathway. After failed attempts to identify a direct binding partner of HQBA with an unmodified and a photocrosslinker-containing analogue of HQBA, genome-wide expression profiles of HQBA-treated MCF7 cells connected the non-structurally related iron chelators ciclopirox and deferoxamine to the HQBA signature in C-Map. The investigators next demonstrated that chelation of intracellular iron, specifically Fe^2+^, is responsible for the biological effects of HQBA; premixing of HQBA with Fe^2+^ completely abrogated its toxicity.

A fourth recent C-Map example is provided by Shaw *et al* (2011). Here, the investigators performed a high-throughput, synthetic lethal screen to identify small molecules that selectively kill mouse embryonic fibroblast (MEFs) expressing oncogenic K-ras. From a collection of >50 000 small molecules, tolperisone was identified as highly selective against K-ras mutant cells compared with wild type. Lanperisone, a tolperisone derivative, demonstrated even more potent activity on K-ras^G212^ mutant cells and was subsequently selected for additional studies. In order to address compound mechanism of action, a lanperisone gene expression signature was derived from MEFs treated for 6 h. As in the prior two examples discussed above, this drug signature was connected to hypoxia and the HIFs by GSEA. However, while HIFs were induced by lanperisone, they did not have a functional role in the selective killing of K-ras mutant cells. In this case, a C-Map query identified numerous small molecules that induce oxidative stress including parthenolide, lomustine, geldanamycin, and 15-*δ*-prostaglandin J2. Induction of reactive oxygen species was then confirmed as the mechanism of lanperisone-induced K-ras selective cytotoxicity.

Although the above studies demonstrate the application of C-Map to connect small molecules with their pathway mechanism of action or direct protein-binding target, the C-Map has also been used for other applications, such as to identify synergistic combinations of compounds as reported by Hassane *et al* (2010). First, the investigators identified parthenolide as a small molecule with activity in both the acute myeloid leukaemia (AML)-initiating cell and the bulk population of AML cells by both inhibiting NF-*κ*B and inducing oxidative stress (Guzman *et al*, 2007). From genome-wide expression profiling of parthenolide-treated AML cells, the investigators noted that a cytoprotective response was induced through activation of the Nrf2 pathway (Hassane *et al*, 2008). They next used the C-Map to identify compounds negatively correlated with a 150-gene parthenolide signature hypothesising that such compounds might counteract the cytoprotective response signature induction and thus diminish the cytoprotective response induced by parthenolide (Hassane *et al*, 2010). PI3K and mTOR inhibitors were among the molecules negatively connected to the parthenolide signature leading to the hypothesis that the PI3K/mTOR pathway is activated in response to parthenolide treatment. The investigators then demonstrated that the PI3K/mTOR pathway is indeed activated by parthenolide using a biochemical approach and that there is synergism with PI3K and mTOR inhibitors in combination with parthenolide *in vitro* and with the pharmacologically superior analogue dimethyl-amino-parthenolide in primary human AML orthotopic models.

In summary, this work demonstrates the power of chemical genomics to enable discovery of modulators of a complex cancer phenotype, identification of the mechanism of action of the small-molecule hits, and nomination of synergistic combinations of compounds. Although this approach is potentially quite powerful, there are limitations. First, the expression readout may be far downstream of the actual binding target and may lead to the pathway of action but not the direct protein target of the molecule. Second, the current build of the C-Map is restricted to a limited number of cell lines and compound doses. Cell context will undoubtedly be important in some cases limiting the ability to identify targets, and the compound doses chosen in C-Map might miss some interactions and be clinically irrelevant in others.

## Genetic screening for protein target identification

Sequencing the human genome and the genome of many model organisms has enabled new opportunity with genetic screening for the identification of protein targets of small-molecule therapeutics. As in the case of the application of gene expression profiling to protein target identification, many of the first proof-of-concept genetic screens were performed in the model organism *Saccharomyces cerevisiae*. An initial study by Giaever *et al* (1999) demonstrated that yeast strains with heterozygous deletion of drug targets can result in sensitisation to the drug target of interest in comparison with the diploid strain (haploinsufficiency profiling). The investigators later scaled this approach to the evaluation of 10 small molecules in 80 genome-wide experiments using a collection of molecularly barcoded heterozygous deletion strains (Giaever *et al*, 2004). Relative abundance of each strain was quantified by amplification of the molecular barcodes from genomic DNA and hybridised to oligonucleotide microarrays containing the complementary barcodes. For many of the compounds evaluated, the most sensitive heterozygous strain carried a deletion of the well validated, direct binding protein target of the molecule. In a publication by Lum *et al* (2004), a genome-wide pool of tagged heterozygotes was used to assess the effects of 78 compounds in *Saccharomyces cerevisiae*. A high-density oligonucleotide array with a two-colour labelling strategy was used to monitor growth rates. In this study, the investigators successfully identified many well-validated targets of small molecules, such as those involving compounds targeting the ergosterol pathway: lovastatin (*HMG1*), terbinafine (*ERG1*), and clotrimazole (*ERG11*). Moreover, they identified new potential targets such as lanosterol synthase as a target of the antianginal drug, molsidomine, and the ribosomal RNA processing exosome as a potential target of the anti-neoplastic agent 5-fluorouracil.

A next important step was the extension of genetic screening to mammalian systems. The production of libraries of DNA-based vectors encoding shRNA targeting most known genes in the human genome now enable genome-wide loss-of-function screens in mammalian cells; furthermore, the generation of libraries of ORFs enable gain-of-function screens. A publication from Burgess *et al* (2008) serves as an example of using a pooled RNAi screening strategy both *in vitro* and *in vivo* to explore the genetic basis of the heterogeneous response to topoisomerase poisons. An *in vitro* screen was performed using retrovirally encoded shRNAs targeting a set of 100 known, or putative, cancer-related genes. The shRNA-containing vectors were transduced into the p19 ^ARF−/−^ E*μ*-Myc murine lymphoma cell line, and infected cells were treated with doxorubicin at doses that typically kill 70–95% of the cells within 24 h. shRNAs enriched after doxorubicin treatment were identified, including shRNAs targeting *p53*, *CHK2*, and one of the known targets of doxorubicin, *TOP2A*. *TOP2A*-directed shRNAs were also confirmed to confer resistance to doxorubicin *in vivo*. Topoisomerase II inhibitors, such as doxorubicin, increase the steady state of the topoisomerase II-DNA cleavage complexes and thus poison the cell with excess complex formation leading to DNA double-strand breaks and apoptosis. Hence, downregulation of *TOP2A* expression would be expected to confer doxorubicin resistance rather than increase sensitivity. Moreover, the investigators demonstrated that shRNAs directed against *TOP2A* cause resistance to another topoisomerase 2 inhibitor, etoposide, but not to the topoisomerase 1 inhibitor camptothecin. In contrast, *TOP1*-directed shRNAs conferred resistance to camptothecin *in vitro* and *in vivo*, and unexpectedly, *TOP1* shRNAs enhanced sensitivity to topoisomerase 2 inhibitors. This study highlights the potential of shRNA-based screening to identify drug targets and determinants of chemotherapy response in mammalian cells.

Parallel to the development of loss-of-function screens, overexpression strategies have been pursued to identify relevant targets of small molecules. Among the first examples of a large-scale overexpression screen for target identification in mammalian cells was a study reported by Luesch *et al* (2006). A total of 27 000 expression cDNAs were screened for suppression of the antiproliferative effect of the cyanobacteria metabolite, aprotoxin A, in the human osteosarcoma cell line U20S. Several cDNA plasmids expressing FGFR protein variants partially rescued the induction of cell cycle arrest and apoptosis induced by aprotoxin A, and phosphorylation of STAT3, a downstream target of FGFR, was inhibited by aprotoxin A. An inverse correlation of FGFR expression with aprotoxin A sensitivity in a panel of cancer cell lines was also observed, suggesting that a highly expressed FGFR pathway is one mechanism of resistance to aprotoxin A in cancer cell lines. There was no inhibition of the kinase activities of FGFR1–FGFR4 by aprotoxin A in biochemical assays, however, and the precise target of apratoxin A in the FGFR pathway remains to be elucidated. Although in this case, a chemogenomic approach did not identify the direct binding target of the small molecule, it enabled identification of a pathway of importance to the mechanism of action of the drug.

More recently, investigators have begun to integrate multiple genetic screens for protein target identification. Hoon *et al* (2008) combined both loss- and gain-of-function chemogenomic strategies for target identification in yeast. In this screening platform, three different yeast pools were interrogated with a single TAG4 array: a homozygous deletion pool, a heterozygous deletion pool, and a pool of genomic library transformants, each treated with a compound of interest. First, a panel of eight reference compounds was evaluated. From the intersection of deletion sensitivity profiling (sensitivity when deleted) and multi-copy suppression profiling (resistance when overexpressed), known targets of methotrexate (*DFR1*), fluconazole (*ERG11*), and rapamycin (*TOR2*) were identified. Next, the investigators extended screening to 188 synthetic chemicals and identified new candidate targets for these molecules. The intersection of deletion sensitivity and multi-copy suppression profiling refined the list of potential targets for subsequent validation studies.

Although genetic screens are another important approach toward target identification, there are potential limitations. First, they are only as good as the library screened. Less comprehensive libraries may miss the critical target of the compound of interest and even comprehensive libraries may not have efficient shRNAs against all of the represented genes or all of the cDNAs expressing fully functional proteins. In addition, shRNAs are well known to have off-target effects, which may confound interpretation of the results and massive overexpression of a gene may not reflect target binding under physiological conditions. Finally, these genetic screens are also limited by one's ability to either infect or transfect the cells of interest.

## Chemical proteomic approaches to protein target identification

In addition to new chemical genomic and genetic approaches to connect small molecules with their protein targets, chemical proteomic approaches have the potential to facilitate protein target identification by cataloguing proteome-wide small molecule–protein interactions using drug affinity chromatography coupled to mass spectrometry (MS) and computational analysis (Rix and Superti-Furga, 2009). In a compound-centric approach to chemical proteomics, the molecule of interest is immobilised on a matrix such that its activity is retained. The cell lysate of interest is then incubated with the affinity matrix and washed before elution. The eluted proteins are then processed by SDS–PAGE or a gel-free approach, digested, and then identified and quantified by MS. One strength of chemical proteomics is the ability to probe the entire proteome, rather than a limited panel of recombinant proteins, and that the small molecules encounter these proteins in their natural state and environment. Another strength is that chemical proteomics can be performed in any cell type or tissue of interest. Disadvantages to this approach are that there is a need for a relatively high quantity of cellular material and that the lysis protocol used may not capture all proteins equally well, particularly membrane-bound proteins. Moreover, a high background level can be created by very abundant proteins or proteins prone to interacting with hydrophobic or charged surfaces. Another challenge is that the identification of binding proteins is semi-quantitative and does not generally provide a tight correlation with half-maximal inhibitory values.

One important advance made in chemical proteomics has been the improvement in quantification via the application of methods such as stable isotope labelling. One successful approach is stable isotope labelling with amino acids in cell culture (SILAC), a metabolic incorporation of a label (e.g., deuterium, ^13^C, ^15^N) into proteins for MS-based quantitative proteomics (Ong *et al*, 2002). This method exploits the ability of live cells to incorporate labelled amino acids through media supplementation. The newly synthesised proteins will incorporate either the ‘light,’ natural isotope abundance forms, or the ‘heavy,’ ^13^C, ^15^N-bearing versions of arginine and lysine amino acids making it possible to monitor quantitative differences at the protein level between conditions. Protein lysates from ‘heavy’ or ‘light’ cultures are incubated with small-molecule-loaded beads versus control beads or small-molecule-loaded beads versus small-molecule-loaded beads and soluble small-molecule competitors. Proteins interacting directly with small molecules are discriminated from nonspecific interaction because of the enrichment in one state over the other and identified by differential ratios in the target pull-down sample (Ong *et al*, 2009).

**Figure 2:**
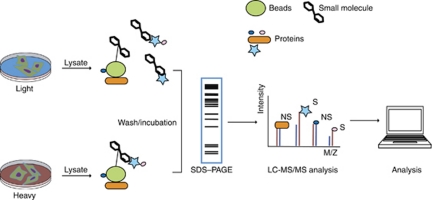
Stable isotope labelling with amino acids in cell culture schema. Adapted from (Ong *et al*, 2009). Two cell populations are grown in medium containing light (blue) or heavy labelled amino acids (red). Protein lysates from the two cell populations are then harvested and incubated with small-molecule-loaded beads or small-molecule-loaded beads and excess of free small molecule to competitively displace target proteins in one of the lysate mixtures. Beads from both lysates are then washed, combined, and boiled. Proteins that remain bound to the immobilised small molecule are eluted, separated by SDS–PAGE, identified and quantified with MS, and SILAC ratios determined. Proteins interacting directly with the small molecule or by secondary interaction with the small molecule (S) will be enriched in the heavy state and will be identified with differential ratios. Nonspecific interactions (NS) will be enriched equally in the light and the heavy states and have ratios closer to 1.

The approach of combining SILAC with small-molecule affinity enrichment was tested by Ong *et al* (2009) to identify targets of kinase inhibitors and immunophilin binders. The investigators compared two experimental designs: one used a bead control and the second used a soluble competition for SILAC target identification. In the first design, the relative abundance of proteins from affinity pull-downs with two different chemically modified bead matrices was tested: small-molecule-loaded beads versus ethanol-loaded beads. In the soluble competition experiment, small-molecule-loaded beads were used in both the heavy and light affinity pull-downs, but in one condition, an excess of small molecule was added to compete for target proteins (Figure 2). To evaluate these approaches, the investigators tested two independent classes of small molecules: kinase inhibitors (R0-31-7549, SB202190, and K252a) and immunophilin binders (AP1497, Pro-AP1497, AP1780, and Pro-AP1780). The investigators concluded that SILAC with small-molecule affinity enrichment improves sensitivity and specificity of unbiased affinity purification-based target identification and that the soluble competition experimental approach is the preferred method in SILAC target identification. In the kinase inhibitor experiments, known protein targets, associated protein complexes, as well as proteins with related biology were successfully identified. In the immunophilin experiments, known targets, such as members of the FKBP family, were identified, and the binding affinities of the FKBP family members with different immunophilins were characterised. Furthermore, methylthioadenosine phosphorylase was identified and validated as a new interactor with all members of the immunophilin series. Although the bead control successfully identified target proteins, it also produced a list of moderate to highly abundant proteins with weak but differential binding to the small molecule. In contrast, the use of the soluble competition design yielded much greater specificity and was largely independent of protein abundance. Moreover, because it uses the same small-molecule affinity matrix in both the control and experiment samples, it circumvents the challenge of selecting an appropriate control matrix.

Although we have focused on SILAC in this review, other stable isotope labelling-based quantitative MS chemical proteomic approaches are in use. Two examples include iTRAQ (isobaric tags for relative and absolute quantification) and ICAT (isotope-coded affinity tag) (Gygi *et al*, 1999; Bantscheff *et al*, 2007a, 2007b; Rix and Superti-Furga, 2009). The iTRAQ technology is a gel-free approach utilising isobaric reagents to label the primary amines of peptides and proteins. The iTRAQ can be implemented after cell or tissue lysis and thus can be used to quantify proteins in any biological system. As such, it is not limited to only those systems that can accommodate incorporation of stable isotopes during cell culture, such as in SILAC. Similarly, ICAT is a gel-free approach that can be used to quantify proteins in any biological system. This approach utilises a chemical reagent that consists of a reactive group for labelling cysteines, an isotopic linker region, and a biotin affinity moiety. The ICAT approach, however, will only quantify proteins with cysteines.

One significant limitation to these affinity-based matrix approaches, however, is the need to derivatise the small molecule for affinity purification with the requirements that the molecule contains a domain, which can be derivatised, that the bioactivity is not altered by the derivitisation, and that the matrix does not impede the binding of the drug to the protein. Alternative approaches to chemical synthesis, such as diversity-oriented synthesis (DOS), are being employed to address this challenge, in part. Here, the goal is to generate collections of molecules with the structural and stereochemical diversity of natural products, which are poised for optimisation and contain functional handles for the generation of affinity reagents (Wang *et al*, 2008). Some successful applications of DOS chemistry led to the identification of the compound robotnikinin as a binder of Sonic Hedgehog and inhibitor of its signalling pathway (Stanton *et al*, 2009), the novel HDAC6 inhibitor tubacin (Haggarty *et al*, 2003), haptamide A as a modulator of Hap3p a subunit of the yeast Hap2/3/4/5p transcription factor complex (Koehler *et al*, 2003), and uretupamine as an activator of a glucose-sensitive transcriptional pathway downstream of Ure2p (Kuruvilla *et al*, 2002).

Alternative chemical proteomics approaches to protein target identification of small molecules, which do not rely on affinity reagents are also under development. For example, SPROX (stability of proteins from rates of oxidation) combines a chemical modification- and MS-based method with MS-based proteomics to measure the thermodynamic stability of protein–ligand complexes (West *et al*, 2010). The SPROX approach makes thermodynamic measurements of protein-folding reactions to detect target protein drug interactions and uses methionine oxidation levels, in complex protein mixtures, to measure transition midpoints consistent with stabilisation. As a proof-of-principle experiment, West *et al* (2010) investigated the protein targets of the immunosuppressive drug cyclosporine A (CsA) in yeast cell lysates. The investigators identified 10 hits that interact with CsA. Two known targets were identified: the direct target cyclophilin A and the indirect target UDP-Glucose-4-Epimerase, as well as 8 previously unreported targets, including proteins involved in glucose metabolism. Although the ability to identify potential direct and indirect drug targets in the absence of an affinity reagent is an advantage, there are disadvantages to the SPROX approach. First, the target must contain a methionine-containing peptide in the domain of the target protein, which has its thermodynamic properties modulated by the small-molecule interaction. Second, there must be sufficiently high protein and ligand concentration to generate a measurable transition midpoint shift. Moreover, the oxidative reaction used to interrogate the folding/unfolding of the proteins might itself alter the protein-folding and ligand-binding properties.

Another chemical proteomic approach to target identification is DARTS (drugs affinity responsive target stability), which exploits the reduction in the protease susceptibility of the target protein on drug binding (Lomenick *et al*, 2009). The DARTS strategy is based on the notion that the binding of a drug to a target protein leads to protein stabilisation or to masking of a protease recognition site, making the protein less susceptible to protease activity. DARTS uses an affinity-based chromatography method that selectively enriches from protein mixtures target proteins that are rendered protease-resistant on small-molecule ligand interaction. In a proof-of-concept experiment, Lomenick *et al* (2009) confirmed by the DARTS approach the previously reported target of several small molecules, such as the immunophilin FKBP12 for the immunosuppressants FK506 and rapamacyin and the human elongation factor-1*α* for the anticancer marine natural product didemnin B. Furthermore, the investigators validated their approach by identifying a new putative target in the yeast cell lysate of the natural product resveratrol, eIF4A. As in SPROX, one major advantage of the DARTS approach is that it does not require derivitising the small molecule; it is not limited by synthetic chemistry and can be applied to any small molecule of interest or natural product. Second, as in SPROX, and unlike cell-based target identification, the DARTS approach is independent of the phenotypic effect of the drug on the system and can thus be performed using any cell type. Like all approaches, however, there are limitations. The binding affinity of the drug to its target might be a limiting feature in DARTS. Second, some proteins might be particularly refractory to protease digestion. Third, drug binding might change the protease susceptibility of non-target proteins.

## Integrating multiple approaches for target identification

Each approach toward target identification has both advantages and limitations as discussed above. To increase the likelihood of correctly identifying target proteins, one might integrate multiple approaches. We employed such a strategy to explore the off-target activities of epidermal growth factor receptor (EGFR) inhibitors in AML (Stegmaier *et al*, 2005). Using GE-HTS, we identified EGFR inhibitors as small-molecule inducers of AML differentiation in AML cell lines and primary patient blasts. However, we were unable to identify EGFR expression in the cell lines responding to these EGFR inhibitors suggesting that in these cell lines, the activity of the EGFR inhibitors was off-target. In order to identify candidate targets of these compounds, we integrated a proteomic and genetic screening approach. A proteomic approach was used with peptide immunoprecipitation-HPLC-MS to identify candidate targets with differential phosphorylation in EGFR inhibitor-treated AML compared with a vehicle control. Spleen tyrosine kinase (SYK), a cytoplasmic tyrosine kinase critical in B-cell development and hematopoietic signalling, was identified as a candidate target. To increase the confidence in hits identified in the proteomic screen, we intersected a high-throughput, lenti-virally delivered shRNA screen designed against the human kinome, which used an AML differentiation signature readout. One of the top scoring shRNAs targeted *SYK*. Subsequent validation studies confirmed that loss of SYK with pharmacological and genetic inhibition impaired AML cell viability and promoted AML cell differentiation *in vitro*. Moreover, the orally available SYK inhibitor, fostamatinib, demonstrated activity in three *in vivo* models of AML, identifying SYK as a new potential druggable protein for AML therapy (Hahn *et al*, 2009). This work demonstrated the power of integrating multiple new approaches to identify the protein target of small-molecule hits emerging from chemical genomic screens.

A similar approach of combining chemogenomics with targeted shRNA screening was taken to identify novel therapeutics for sickle cell disease. (Bradner *et al*, 2010) Here, the investigators developed a GE-HTS assay to measure the expression of globin genes during erythroid differentiation. They next performed a small-molecule library screen and identified histone deacetylase (HDAC) inhibitors as increasing the *γ*/*β* ratio. To confirm the dependency of the induced phenotype on HDAC inhibition and to further delineate the critical HDACs, the investigators genetically targeted individual HDAC transcripts with shRNAs. Histone deacetylase-1 and 2 were validated as potential targets mediating the induction of fetal haemoglobin in primary erythroid cells.

## Conclusion

The development of new approaches to small-molecule library screening enables opportunity for compound discovery to target pharmacologically intractable proteins, such as transcription factors, and to modulate biological processes in the absence of knowledge of the target critical to the state switch. With the parallel development of alternative strategies for protein target identification, including chemical genomic, genetic, and chemical proteomic, the potential to understand both the on-target mechanisms of action of these newly discovered molecules as well as to predict the potential toxicities becomes feasible. The efficient execution of these projects involves significant infrastructure and expertise in multiple disciplines including chemistry, genomics, proteomics, and bioinformatics. Only with collaborative efforts across academic institutions and industry will the potential of these approaches be fully realised and translated to more effective therapies for patients with cancer.

## Figures and Tables

**Figure 1:**
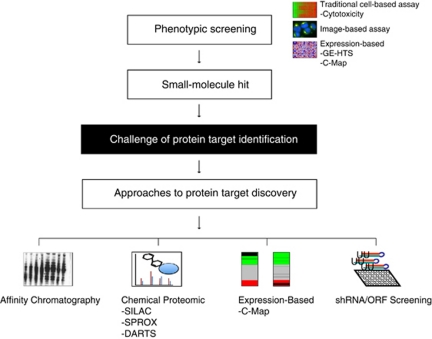
Approaches to target identification. One of the challenges of phenotypic screening is the identification of the protein target(s) of the compound hit. Although an affinity chromatography-based approach was once among the only solutions to this challenge, new chemical, proteomic, gene expression, and shRNA screening-based approaches are increasing the armamentarium of tools for identifying the mechanism of action of compounds emerging from phenotypic screens.
